# Psychiatric polygenic risk as a predictor of COVID-19 risk and severity: insight into the genetic overlap between schizophrenia and COVID-19

**DOI:** 10.1038/s41398-023-02482-7

**Published:** 2023-06-06

**Authors:** M. Alemany-Navarro, S. Diz-de Almeida, R. Cruz, J. A. Riancho, A. Rojas-Martínez, P. Lapunzina, C. Flores, Scourge Cohort Group, A. Carracedo

**Affiliations:** 1 IBIS (Universidad de Sevilla, HUVR, Junta de Andalucia, CSIC), Sevilla, Spain; 2 Centro Singular de Investigación en Medicina Molecular y Enfermedades Crónicas (CIMUS), Universidade de Santiago de Compostela, Santiago de Compostela, Spain; 3 Fundación Pública Galega de Medicina Xenómica, Sistema Galego de Saúde (SERGAS) Santiago de Compostela, Santiago de Compostela, Spain; 4 Grupo de Genética. Instituto de Investigación Sanitaria de Santiago (IDIS), Santiago de Compostela, Spain; 5 Centro de Investigación Biomédica en Red de Enfermedades Raras (CIBERER-ISCIII), Instituto de Salud Carlos III, Madrid, Spain; 6 IDIVAL, Cantabria, Spain; 7 Universidad de Cantabria, Cantabria, Spain; 8 Hospital U M Valdecilla, Cantabria, Spain; 9 Tecnologico de Monterrey, Escuela de Medicina y Ciencias de la Salud, Monterrey, Mexico; 10 Instituto de Genética Médica y Molecular (INGEMM) del Hospital Universitario La Paz, Madrid, Spain; 11 ERN-ITHACA-European Reference Network, Santa Cruz de Tenerife, Canarias, Spain; 12 Research Unit, Hospital Universitario N.S. de Candelaria, Santa Cruz de Tenerife, Spain; 13 Centro de Investigación Biomédica en Red de Enfermedades Respiratorias, Instituto de Salud Carlos III, Madrid, Spain; 14 Genomics Division, Instituto Tecnológico y de Energías Renovables, Santa Cruz de Tenerife, Spain; 15 Department of Clinical Sciences, University Fernando Pessoa Canarias, Las Palmas de Gran Canaria, Spain

**Keywords:** Predictive markers, Clinical genetics, Schizophrenia

## Abstract

Despite the high contagion and mortality rates that have accompanied the coronavirus disease-19 (COVID-19) pandemic, the clinical presentation of the syndrome varies greatly from one individual to another. Potential host factors that accompany greater risk from COVID-19 have been sought and schizophrenia (SCZ) patients seem to present more severe COVID-19 than control counterparts, with certain gene expression similarities between psychiatric and COVID-19 patients reported. We used summary statistics from the last SCZ, bipolar disorder (BD), and depression (DEP) meta-analyses available on the Psychiatric Genomics Consortium webpage to calculate polygenic risk scores (PRSs) for a target sample of 11,977 COVID-19 cases and 5943 subjects with unknown COVID-19 status. Linkage disequilibrium score (LDSC) regression analysis was performed when positive associations were obtained from the PRS analysis. The SCZ PRS was a significant predictor in the case/control, symptomatic/asymptomatic, and hospitalization/no hospitalization analyses in the total and female samples; and of symptomatic/asymptomatic status in men. No significant associations were found for the BD or DEP PRS or in the LDSC regression analysis. SNP-based genetic risk for SCZ, but not for BD or DEP, may be associated with higher risk of SARS-CoV-2 infection and COVID-19 severity, especially among women; however, predictive accuracy barely exceeded chance level. We believe that the inclusion of sexual loci and rare variations in the analysis of genomic overlap between SCZ and COVID-19 will help to elucidate the genetic commonalities between these conditions.

## Introduction

The severe acute respiratory syndrome or coronavirus disease-19 (COVID-19) caused by the novel coronavirus SARS-CoV-2 (severe acute respiratory syndrome coronavirus 2) turned into the worst global health problem during 2020 and 2021 given the rapid spread of the virus, and the severity and mortality of the syndrome. After recognition of COVID-19 as a pandemic by the World Health Organization (WHO) on March 11, 2020, researchers all over the world have attempted to characterize the syndrome’s epidemiology and to identify aggravating factors that may be highly diverse in nature.

Despite the high contagion and mortality rates, not everyone exposed to SARS-CoV-2 is affected the same way, with some people even presenting no symptoms at all after infection. Growing evidence points to certain groups being at higher risk of a severe COVID-19 outcome, such as the elderly, people with previous health conditions, men, and those with a high body mass index [1–5]. Thus, the identification of individual characteristics that correspond with increased risk is one of the greatest and most important challenges in COVID-19 research.

Certain previous clinical comorbidities that have been linked to worse COVID-19 outcomes and persistent or long COVID-19, such as cardiovascular disease (CVD), respiratory diseases, metabolic disorders, gastrointestinal symptoms, systemic inflammation, or autoimmunity, are often reported among psychiatric probands [6–12]. However, evidence on the prevalence of COVID-19, just as of COVID-19 severity and outcome, among psychiatric patients is scarce. Some authors have reported increased prevalence of COVID-19 among schizophrenia (SCZ) patients, with a higher fatal outcome frequency in 65- to 80-year-old patients, and higher intensive care unit (UCI) admission rates in patients younger than 55, than in non-psychiatric COVID-19 patients in the same age ranges [7, 8, 13].

Although not meeting the criteria for the abovementioned medical conditions that accompany a greater risk of COVID-19, the immunological dysfunctions usually reported in psychiatric disorders may increase vulnerability to COVID-19 among this population. SCZ, major depressive disorder (MDD) and bipolar disorder (BD), among other conditions, have been associated with a persistent inflammatory state, with increased pro-inflammatory markers, such as C-reactive protein, CRP; interleukin (IL)-1β and IL-6; tumor necrosis factor (TNF)-α; transforming growth factor (TFG)-β; interferon (IFN)-γ; or vascular endothelial growth factor (VEGF) [14–22]. Impaired adaptive immunity and autoimmunity have also been reported in these three psychiatric conditions [23–28].

Meanwhile, COVID-19 has been found to directly or indirectly induce the abovementioned medical conditions and also pathophysiological mechanisms reported to be more prevalent among psychiatric subjects than healthy controls (such as chronic systemic inflammation and neuroinflammation, microbiome disruptions, or increased oxidative stress) [9–11, 29–35]. Furthermore, certain medications used in the treatment of psychosis or affective disorders have been proved to be useful in preventing neurotropic adverse events caused by SARS-CoV-2 [36, 37]. However, despite the pathophysiological parallels observed in certain psychiatric disorders and COVID-19, we do not know to what extent they might be due to shared genetic risk factors.

Potential genetic host factors that accompany greater risk from COVID-19 have been sought via such initiatives as the COVID-19 Host Genetics Initiative (COVID-19 HGI), an international research consortium that studies the role of human genetics in SARS-CoV-2 infection and COVID-19 disease response. Besides the implication of *ACE2* and *TMPRSS2* (which code for the main proteins involved in SARS-CoV-2 infection) stemming from the results of candidate gene and gene expression studies [38–41], genome-wide association studies (GWASs) have reported consistent evidence on the involvement of a cluster of genes in the 3p21.31 region (*SLC6A20*, *LZTFL1*, *CCR9*, *FYCO1*, *CXCR6* and *XCR1*), with *SLC6A20* having been proposed as a candidate gene for this region; and the *AB0* blood type locus at chromosome 9 (9q34.2) [42–46]. In silico analysis [47] has also implicated variations of the major histocompatibility complex (MHC) class I genes (human leukocyte antigen (HLA) genes) in susceptibility to and severity of COVID-19 [47].

Genetic approaches to the study of psychiatric disorders have associated B-lymphocyte activity and MHC loci with SCZ and BD through non-synonymous, untranslated variant regions (UTR), and gene enhancer variants [48–50]; and genetic variants involved in inflammatory pathways have been reported as sources of convergence in the etiology of depression (DEP), BD, and SCZ [51]. Beyond risk involving genes with an immune function, others with different biological functions have been identified as common risk factors for COVID-19 and different psychiatric disorders. Analysis of GWASs and whole-genome sequencing (WGS) data in COVID-19 and psychiatric patients revealed 20 and 32 genes, out of 146 significant COVID-19 genes, to be associated with BD and SCZ, respectively [52]. In addition, analysis of peripheral blood mononuclear cell (PBMC) transcriptomes in these three conditions revealed 39 and 22 dysregulated genes in COVID-19 patients, and in BD and SCZ, respectively, as well as multiple shared biological pathways and processes after enrichment analysis. Furthermore, COVID-19 could result in the perturbed expression of genes involved in SCZ and BD [52].

In this study, we construct polygenic risk scores (PRSs) from SCZ, BD, and DEP risk alleles and test their predictive ability for COVID-19 susceptibility and severity. Given that different biological functions that go beyond brain function and even the immune system [53–55] have been implicated in the pathophysiology of neuropsychiatric disorders, and that some well-characterized genes may have functions that as yet remain unknown, we analyzed the predictive capability of global PRSs that gathered together common variants across the whole genome. We also analyzed the predictive power of a more specific PRS, built from variants of genes with known immune function when significance was reached in the global PRS analyses. We expected to find that both the global and immune PRSs predict SARS-CoV-2 infection and COVID-19 severity, symptomatology, and need for hospitalization.

## Methods and materials

Detailed information on recruitment and the genotyping and quality control procedures are described elsewhere [56].

### Subjects—target sample

Our COVID-19 cohort comprised 11,977 subjects with a positive diagnosis for COVID-19 (PCR-based test or local clinical and laboratory procedures) from 34 Spanish hospitals in 25 different cities (See Table S1 in Cruz et al. [56] for a list of hospitals or research centers with their respective samples). All the hospitals formed part of the Spanish COalition to Unlock Research on host GEnetics on COVID-19 (SCOURGE). The study samples and data were collected by the participating centers through their respective biobanks after informed consent. The whole project was approved by the Galician Ethical Committee, ref.: 2020/197. An additional 5943 people with unknown COVID-19 status were included as the control sample: 3437 from the Spanish DNA biobank (https://www.bancoadn.org) and 2506 samples from the GR@CE consortium [57]. Study data were collected and managed using the REDCap software at the *Centro de Investigación Biomédica en Red* (CIBER) [58, 59].

### Genotype data, quality control and imputation

Details of the genotyping of the samples, quality control procedures and imputation process are described in the main study [56]. Given that imputation was conducted based on the TOPMed version r2 reference panel (GRCh38) [60], data were realigned to the version GRCh37 in order to match it with the discovery sample data using the UCSC Genome Browser [61] (http://genome.ucsc.edu). We further removed imputed variants with a call rate < 98%.

### Polygenic risk score (PRS) analysis

In the present study we followed a polygenic scoring approach. Briefly, a PRS is a weighted sum of the risk alleles associated with a trait that an individual carries and provides an estimate of the genetic liability for that given trait. Effect sizes for each SNP are retrieved from GWAS summary statistics of a discovery sample and are used to build the PRS, which is applied to a target sample for which genotype data is available. Here, PRS were built for SCZ, BD and DEP using summary statistics from the PGC [50, 62–64] (discovery sample) and applied to our COVID-19 cohort [56] (target sample), resulting in a score for each individual and trait. From these scores we identified which individuals were at a higher or lower genetic risk of developing either SCZ, BD or DEP and explored whether this genetic liability was associated to a higher risk of developing severe COVID-19 disease.

### Composition of the polygenic risk scores—discovery sample

The discovery sample for SCZ PRS estimation included those individuals with European ancestry from the Waive 3 Schizophrenia Meta-analysis [62], which constitutes the SCZ GWAS data most recently uploaded to the Psychiatric Genomics Consortium (PGC) webpage (https://www.med.unc.edu/pgc/download-results/). The Waive 3 Schizophrenia Meta-analysis summary statistics were downloaded from the PGC webpage. European individuals accounted for approximately 80% of the total sample of 161,405 unrelated individuals from 90 different cohorts (67,390 patients with SCZ or schizoaffective disorder and 94,015 healthy controls) [62]. A full clinical characterization of the Waive 3 SCZ sample can be seen in the supplementary material of Trubetskoy et al. [62] (Supplementary Information; *Case-control sample descriptions*).

For the BD PRS, the discovery sample was formed of 57 BD cohorts recruited in Europe, Australia, and North America consisting of 41,917 cases diagnosed with type I or type II BD and 371,549 controls of European descent. The summary statistics were downloaded from the PGC webpage and corresponded to the results of the largest GWAS and most recent meta-analysis of BD [50]. A description of the BD samples can be seen in the supplementary material of Mullins, et al., 2021 (Supplementary Note, *Sample Descriptions*) [50].

The discovery sample for the DEP PRS was formed of 33 cohorts from the Psychiatric Genomics Consortium (excluding the UK Biobank and 23andMe data) [63] and the broad DEP phenotype in the full release of the UK Biobank [64]. The total sample was formed of 246,363 cases and 561,190 controls. DEP summary statistics were also downloaded from the PGC webpage. Clinical description of the samples studied in the published DEP GWAS can be seen in Howard et al. [64] (“Methods” and Supplementary information, Supplementary Note 2).

Using the summary statistics from the GWASs of the three psychiatric disorders, we derived PRSs using the PRS-CS software [65], which uses all SNPs in a Bayesian framework, inferring posterior effect sizes under continuous shrinkage (CS) priors, making use of a linkage disequilibrium (LD) reference panel, in this case the 1000 Genomes Project LD reference panel for European ancestry (https://github.com/getian107/PRScs). Instead of setting a specific shrinkage parameter, we carried out a grid search (e.g., phi = 1e−6, 1e−4, 1e−2, 1) to find the most appropriate phi value for the dataset, as recommended in the software manual to improve the predictive performance. We set 10,000 as the number of Markov chain Monte Carlo (MCMC) iterations, and the default values for the remaining parameters.

### Polygenic risk score (PRS) statistical analysis

#### Global PRSs

Logistic regression models were used to analyze the capacity of the calculated PRSs to predict SARS-CoV-2 infection (*RISK* analysis), the presence or absence of symptoms (*SYMP*), having been hospitalized or not (*HOSP*), and critical status (*CRIT*). Given that COVID-19 severity was assessed in the cases using a five-level severity scale (0–4), as described elsewhere (see Table 1 in Cruz et al. [56]), cases were considered critical if their severity score was 4. We added sex, age, and the first 10 principal components as adjustment variables in the models. To compare the variance proportions explained by the different PRSs we estimated a pseudo R2 on the liability scale, as described in Lee et al. [66]. The prevalence of cases and symptomatic cases were considered to be 9.9% and 6.9%, respectively, as reported at the end of 2020 by the Spanish government (https://www.mscbs.gob.es/gabinetePrensa/notaPrensa/pdf/15.12151220163348113.pdf). The prevalence of both hospitalization and critical status were considered to be 0.5% [56]. These analyses were performed with the R package, version 4.0.2. The same analyses were performed after stratifying by sex, including age and the first 10 principal components as adjustment variables.

Logistic regression analysis was also performed classifying the PRSs in deciles to estimate the corresponding odds ratio (OR) considering a confidence interval (CI) of 95%.

We estimated the area under the receiver operating characteristic (ROC) curve (the AUC) to evaluate the performance of the PRSs as predictors of each of the dependent variables by comparing the AUC when including and excluding the PRSs in the covariate model. OR and AUC were calculated when significance was reached after applying a multiple comparison correction strategy.

#### Immune PRS

Given the growing evidence of immune dysfunction playing a role among psychiatric patients, we calculated a PRS constructed from variants of genes with an established immune role when associations had been obtained with the global PRSs. In this way we aimed to narrow down the genetic burden of the PRS and analyze whether immune-related variants were responsible for the observed prediction. We used MAGMA v1.08b to annotate the SNPs in common between the SCOURGE data and the SCZ/DEP/BD summary statistics, and selected those SNPs in genes that are involved in immune pathways according to *KEGG pathways* (Kyoto Encyclopedia of Genes and Genomes; https://www.genome.jp/kegg/pathway.html#organismal). Five kb upstream and downstream windows were added to the start and end of these immune-related genes. The same analyses described above were performed for the resultant immune PRS.

We applied a multiple-comparison correction considering the 4 tests (*RISK*, *SYMP*, *HOSP*, and *CRIT*,) and 3 PRSs (SCZ, BD, and DEP): 0.05/12 = 0.004. Given that some of these tests overlapped in some way (*RISK* and *SYMP*: a large proportion of cases were symptomatic; *HOSP* and *CRIT*: a large proportion of critical cases were hospitalized), we chose a rather conservative approach when correcting for significance.

### Genetic correlation (r_G_) analysis: linkage disequilibrium score (LDSC) regression

We performed post-hoc LD score (LDSC) regression analysis [67] in order to estimate the correlation between the genomic risks for each of the three psychiatric disorders and the COVID-19 variables when significance was reached in the analysis of any of the three global PRSs. The purpose of this was to seek confirmation of the results obtained by PRS analysis through a different approach, assessing the genomic risk overlap between different phenotypes [68].

## Results

### Sample, genotype and imputed data

The final target sample was formed by 15,045 individuals: 9371 COVID-19 positive cases (5028 female, 53.7%; mean age = 62.6) and 5674 individuals with unknown COVID-19 status added as a control population (2752 female, 46.3%; mean age = 53.1). The sociodemographic and clinical characteristics of the SCOURGE sample can be seen elsewhere (Table 2 in Cruz et al. [56]). After imputation and QC, 6,317,562 genetic markers were kept.

### Polygenic risk scores

#### Schizophrenia (SCZ)

**Fig. 1:**
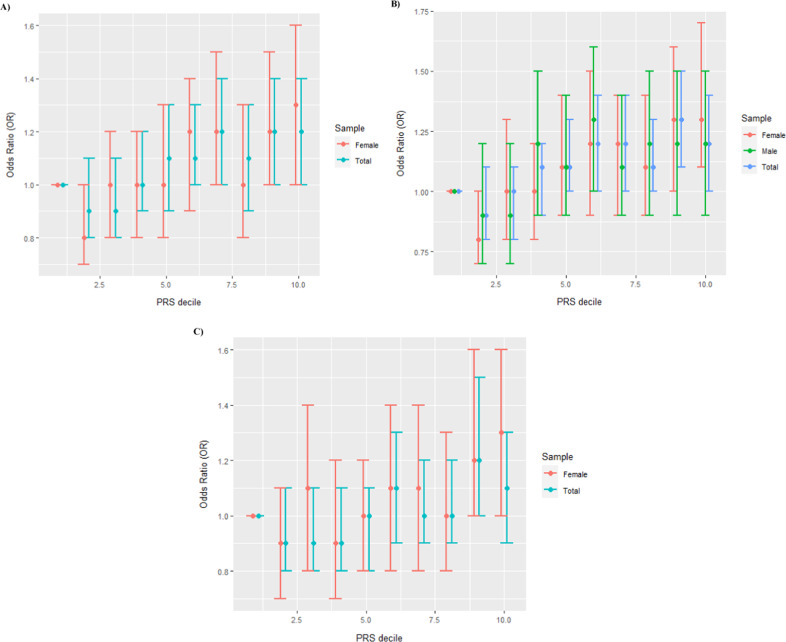
Odds ratios (OR) by the decile of the global SCZ PRS. OR for **A** COVID-19 cases; **B** symptomatic COVID-19 cases; and **C** hospitalized COVID-19 cases. Error bars represent 95% confidence intervals.

The global PRS was constructed from 708,399 variants. Table 1 shows the results of the analyses in which significance was reached (*RISK*, *SYMP*, and *HOSP, p* < 0.004). The associations found in the total sample were maintained in the female sample, while only *SYMP* analysis was significant in the male sample. The ORs by the decile of the estimated PRS for case/control, symptomatic/asymptomatic and hospitalization/no hospitalization are shown in Fig. 1. The AUC did not vary when comparing covariate models with and without PRS in the following cases: total sample (*RISK* AUC = 73.2%/73.2%; *SYMP* AUC = 72.3%/72.2%; *HOSP* AUC = 81.4%/81.4%); female sample (*RISK* AUC = 71.3%/71.2%; *SYMP* AUC = 70.0%/69.8%; *HOSP* AUC = 80.8%/80.8%); or male sample (*SYMP* AUC = 77.1%/77.1%).

**Table 1:** Results of the global SCZ PRS analysis in the total and sex-stratified samples.

*β*	SE	*P*	R2	PRS R2
Total sample				
*RISK (N* = *15,047; 5674 controls and 9373 cases)*				
96,906.49	26,991.08	3.30E−04*	0.199	0.002
*SYMP (N* = *14,885; 6295 controls and 8590 cases)*				
125,266.50	26,527.47	2.33E−06*	0.170	0.002
*HOSP (N* = *15,024; 9056 controls and 5968 cases)*				
100,892.5	29,053.52	5.15E−04*	0.417	6E−04
Female sample				
*RISK (N* = *7658; 2630 controls and 5028 cases)*				
128,303.2	38,597.79	8.87E−04*	0.182	0.001
*SYMP (N* = *7658; 3073 controls and 4483 cases)*				
154,087.1	37,285.84	3.59E−05*	0.148	0.007
*HOSP (N* = *7645; 5115 controls and 7645 cases)*				
127,810.40	42,244.92	2.48E−03*	0.397	0.001
Male sample				
*RISK (N* = *7387; 3044 controls and 4343 cases)*				
83,947.24	39,023.89	0.032	0.275	0.003
*SYMP (N* = *7387; 3222 controls and 4105 cases)*				
115,629.3	38,890.53	0.003*	0.273	0.005
*HOSP (N* = *7377; 3941 controls and 3436 cases)*				
84,937.02	40,377.29	0.035	0.280	−6E−04

*β* beta value, *SE* standard error, *R2* Nagelkerke R2 on the liability scale for the covariate model, *PRS R2* Nagelkerke R2 on the liability scale for the PRS (PRS predictive performance).

Asterisks (*) represent significance after multiple-comparison correction (*p* < 0.004).

Since significant associations were found with the global SCZ PSR, in this case variants of genes involved in different immune pathways were selected to construct the immune PRS. One thousand and ten genes involved in immune pathways (*KEGG* pathways) were present in the discovery and target sample data. The immune PRS was built from 7,375 variants. No significant association was reached in the total or sex-stratified samples.

#### Bipolar disorder (BD)

The global BD PRS was formed from 707,450 variants. No analysis with the global BD PRS reported significant results.

#### Depression (DEP)

The global DEP PRS was formed from 699,966 variants. No significant associations were obtained.

### Linkage disequilibrium score (LDSC) regression analysis

Given that the SCZ PRS was the only one for which associations were obtained, we performed LDSC regression analysis for those variables predicted by the SCZ PRS in the total sample. We considered the GWAS results of COVID-19 case/control and hospitalization/no hospitalization analyses [56]. Although significant associations were found for the SCZ PRS in the *SYMP* analysis, symptomatic/asymptomatic status was not analyzed in the main GWAS [56], thus we did not have results for this analysis. The analyses were also performed with the summary statistics from the last COVID-19 HGI GWAS meta-analysis (https://www.covid19hg.org/results/r7/). LDSC regression analyses did not report significant genetic correlations between SCZ risk and COVID-19 case/control or hospitalization/no hospitalization with the summary statistics from Cruz et al. [56] or COVID-19 HGI GWAS meta-analysis (See Supplementary Table 1). Given the lack of significance in the LDSC regression analyses, performing Mendelian randomization for COVID-19 and SCZ genetic risks was not justified.

## Discussion

In this study we analyzed the ability of PRSs built from SCZ, BD, and DEP risk alleles to predict SARS-CoV-2 infection susceptibility, the presence/absence of symptomatology, being hospitalized or not, and COVID-19 critical status. We obtained significant predictions for the global SCZ PRS in the case/control (*RISK*), symptomatic/asymptomatic (*SYMP*), and hospitalization/no hospitalization (*HOSP*) analyses in the total sample, all of which were maintained in the female only sample. Significant results were obtained in the *SYMP* analysis in men. No significant associations were found for the immune SCZ PRS, or the global BD or DEP PRSs. The LDSC regressions revealed a non-significant trend towards genetic correlations between SCZ risk and COVID-19 case/control and hospitalization/no hospitalization.

The loss of significant associations for the immune SCZ PRS as compared to the global SCZ PRS seems to point to a loss of statistical power when considering only variants of immune-related genes, and thus the involvement of a wider range of functional categories with regards to the genetic overlap between SCZ and COVID-19. Besides the immune dysfunction found in SCZ patients [15, 23] and the association of SCZ risk with immune-related genetic loci [48, 69], multiple medical conditions and biological abnormalities that go beyond immune function may be shared between SCZ [9, 33–35, 70, 71] and COVID-19 patients [30, 72]. Transcriptomic markers have been reported to be shared between COVID-19 and SCZ, implicating the dysregulation of 22 genes (only two of which are immune related) and multiple biological pathways [52]. More importantly, 32 genes involved in a variety of biological systems have been genome-wide associated (*P* < 1E−06) with both COVID-19 and SCZ in WGS and GWAS results [52].

We expected to obtain positive results in the LDSC regression analysis for SCZ and COVID-19 given that PRS associations had been found, and considering the genetic correlation between COVID-19 and SCZ suggested by Moni et al. [52]. However, those authors analyzed the overlap of differentially expressed genes in COVID-19 and psychiatric patients, not the correlation of their SNP-based risks. In addition, although both PRS and LDSC regression analyses look for similarities in the genetic architecture associated with two disorders, their approaches are qualitatively different. This is particularly relevant in our study, where the effect sizes used in the estimation of the different PRSs were posterior effect sizes (see Methods), instead of the original ORs from the summary statistics, which are those used in genetic correlation analyses. Divergent results have been found for PRS and genetic correlation analyses even when both traits studied were psychiatric. As an example, although BD and SCZ have been reported to be genetically correlated, an SCZ PRS does not seem to predict first-episode psychosis among BD patients [73]. Thus, although we cannot conclude that there is a SNP-based genetic correlation between COVID-19 case/control or hospitalization/no hospitalization, and SCZ risk, a polygenic score constructed from SCZ weighted risk common variants is capable of predicting these COVID-19 variables together with symptomatic/asymptomatic status.

Although SNP-based risk for SCZ seems to be identical for females and males, this is not the case for COVID-19 risk and severity, which appear to have greater genetic burden in males [56]. Thus, we decided to stratify the PRS analyses by sex. The associations found for the global SCZ PRS (*RISK*, *SYMP* and *HOSP*) were maintained in the female sample and lost in males, except for the *SYMP* analysis. These findings could be partly explained by the greater presence of pleiotropic genetic risk variants for different clinical conditions in female patients already suggested [74–76]. The parity reached in the epidemiology of different psychiatric and neurologic disorders in adulthood in comparison to the disparity found in childhood for a variety of psychiatric disorders, where prevalence is greater in males during childhood, suggests that different factors and mechanisms may be involved in the pathophysiology of different disorders in females and males [77, 78]. Some X and Y chromosome-linked loci have been proposed as mediators of the sex-based differences in SCZ risk [79, 80]. However, the X chromosome was not accounted for in our PRSs, thus we cannot know if the inclusion of X-linked risk alleles would have made any difference to our results. Beyond sex chromosomes, the results of some genomic studies suggest that males may be protected from the development of certain psychiatric conditions: they may require a greater genetic burden to express a given clinical phenotype [74–76]. In line with this, different biomarkers involved in gene expression regulation have been reported for female and male SCZ patients [81, 82] and epidemiological studies have also noted higher prevalence of neurologic and somatic comorbidities among female SCZ patients [83–86].

Contrary to what we expected, different results were obtained for the different psychiatric disorders. We especially expected to find similar results for SCZ and BD, considering the commonalities reported in their genetic risks and the high SNP-based genetic correlation and coheritability between the conditions (*r*_G_ = 0.68 ± 0.04) [48–51, 87–89]. However, disorder-specific genetic loci have been proposed, and different phenotypes have been correlated with SCZ and BD [87, 90]. In addition, greater genetic similarities between SCZ and BD have been found when psychotic symptoms were present in the latter, which is in accordance with the suggestion that different BD subtypes have different biological underpinnings [91]. In accordance with this last notion, we think that the study on the genetic overlap between BD subtypes with psychosis and COVID-19 may reveal different results than those presented in this study.

From our findings with the DEP PRS, we cannot conclude that the risk for DEP predicts SARS-CoV-2 infection vulnerability, or COVID-19 symptomatology or severity. It is noteworthy that, in addition to a relatively low heritability of MDD (28–44% [92]) when compared to SCZ or BD, part of the DEP discovery sample was comprised of individuals with a self-reported broad DEP phenotype, in contrast to individuals diagnosed with MDD by experts following international diagnostic manuals [93]. We think that the use of a discovery sample in which cases were only patients diagnosed with MDD, which may represent rather endogenous, biologically-determined forms of DEP, would facilitate the identification of PRS associations.

Despite the significant associations with SCZ passing multiple-comparison correction, the inclusion of the SCZ PRS in the adjusted models (sex + age+ principal components) did not involve any change in the estimated AUCs (see Supplementary Fig. 1). However, in previous research even BD PRS models applied to SCZ and MDD samples barely surpassed chance discrimination levels (AUC = 0.56 and 0.55, respectively [94]). Thus, given that quite different phenotypes are being analyzed in the present study (COVID-19 and psychiatric disorders), our main purpose was not to validate predictive models for SARS-CoV-2 infection or COVID-19 syndrome, but rather to analyze possible overlap between common variations underlying SCZ/BD/DEP and COVID-19 that could be further explored in the future. The fact that the prediction obtained for case/control, symptomatic/asymptomatic, and hospitalization/no hospitalization—the last involving an intermediate severity level between symptomatic and critical—was lost in the comparison of critical and non-critical status may be due to admission to the intensity care unit, need for mechanical ventilation, or fatal outcome after hospitalization depending more on disease-specific genetic factors, in addition to non-genetic ones such as age [56, 95].

One of the limitations of our study is the lack of information regarding the possible use of psychotropics in our sample, given their possible influence on COVID-19 response, such as the evidenced protective effect of diverse antidepressants against COVID-19, and an apparent greater risk of COVID-19 infection associated with the use of some antipsychotics [96, 97]. In addition, although the status for a variety of comorbid medical conditions was collected for our COVID-19 cohort, we did not have information regarding psychiatric diagnosis. Having information about psychiatric comorbidities in our COVID-19 cohort would have enable statistically controlling for psychiatric status, or performing stratified analyses for psychiatric status.

In this study, we decided to use a PRS approach, which we consider was the most powerful method for our goal, since it permits us to account for genomic markers with small effects and to analyze genomic overlap that may go unnoticed by looking at GWAS results, given the difficulties for GWASs to identify associated loci [65, 98]. Looking at our significant findings with the global SCZ PRS, there may be shared SNP-based genetic risk between SCZ and COVID-19, and this would, at least partly, explain the greater COVID-19 prevalence and severity reported among SCZ patients [7, 8, 13]. Given that GWAS results for women and men are reported to be almost identical in SCZ, the differences found in the prediction capability of the PRS in this study must be due to the sex-based differences in the genetic risk for COVID-19 previously reported [62]. Considering previous findings concerning certain overlap between gene expression profiles of COVID-19 and those of psychiatric patients, including SCZ [52], and the important role of inherited and rare de novo variants in SCZ and COVID-19 [99–101], further research on genetic risk overlap between these conditions, including the analysis of rare variation from DNA sequencing approaches, will offer more insight into the greater risk that SCZ patients may have for COVID-19 risk and severity. Moreover, the inclusion of sexual chromosomes in the analysis will further inform about sex-based differences in the genetic risk shared by the two conditions.

## Supplementary information

Supplemental material

## Data Availability

Summary statistics of the data of the main study [56] has been aggregated with those from the COVID-19 Host Genetics Initiative (https://www.covid19hg.org); results of this study will be shared upon request to the corresponding author.

## Code availability

All the analysis software used is open source (PLINK 1.9, TopMed Imputation Server version r2, PRS-CS software, MAGMA v1.08b, LDSC (LD SCore) v1.0.1). No custom algorithms or software were used.
