# Supplementary material for: Psychiatric polygenic risk as a predictor of COVID-19 risk and severity: insight into the genetic overlap between schizophrenia and COVID-19
Source: Transl Psychiatry. 2023 Jun 6;13:189. doi: 10.1038/s41398-023-02482-7 (PMC10243274; doi:10.1038/s41398-023-02482-7)
Supplement: Supplementary file 1 — Supplemental material [file 41398_2023_2482_MOESM1_ESM.pdf]

Table S1. Results of the LDSC regression analysis for SCZ risk and COVID-19 risk and hospitalization.

	SCZ h2 on the observed scale	***COVID-19 h2 on the observed scale***	***rG mean***	***P***
	**Case/control status**			
Cruz et al. (2022) [56]	0.365 ± 0.014	0.046 ± 0.037	0.170 ± 0.137	0.215
COVID-19 HGI	0.365 ± 0.014	0.053 ± 0.010	0.006 ± 0.042	0.879
	**Hospitalized/non-hospitalized status**			
Cruz et al. (2022) [56]	0.366 ± 0.014	0.081 ± 0.036	0.089 ± 0.087	0.305
COVID-19 HGI	0.366 ± 0.013	0.307 ± 0.064	0.047 ± 0.031	0.121

SCZ, schizophrenia; *h2 on the observed scale*, heritability on the observed scale (mean ± SD); *rG*, genetic correlation.

A)

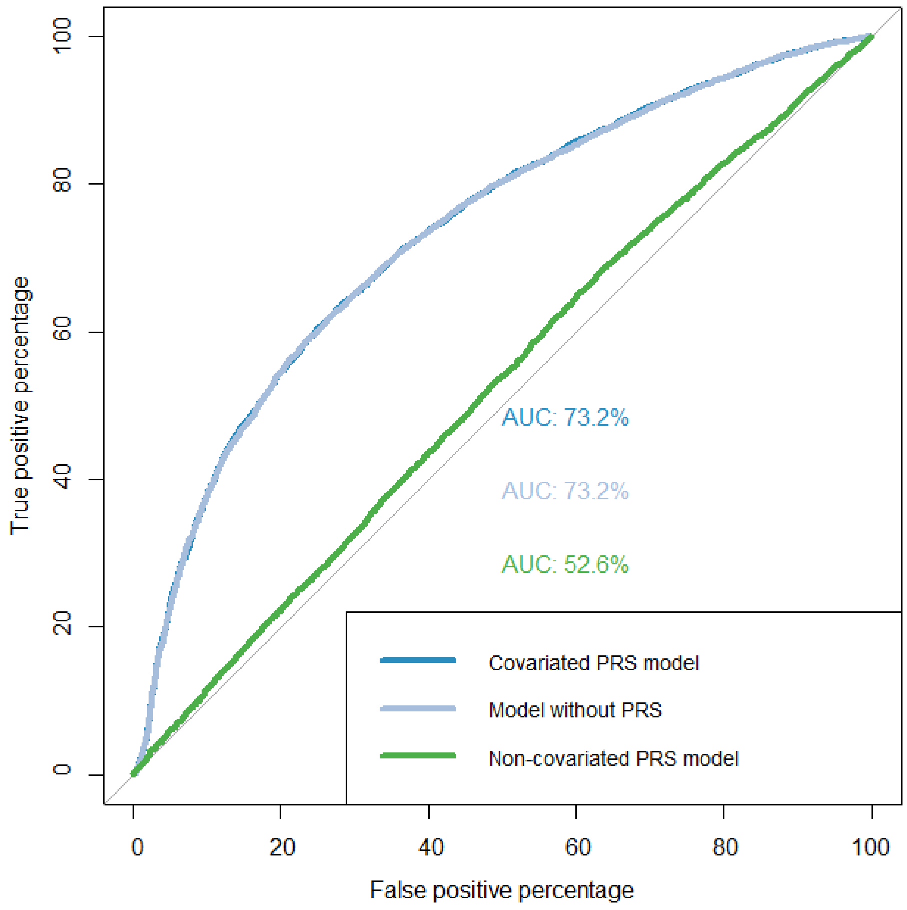

True positive percentage
0
20
40
60
80
100
False positive percentage
AUC: 73.2%
AUC: 73.2%
AUC: 52.6%
Covariated PRS model
Model without PRS
Non-covariated PRS model

B)

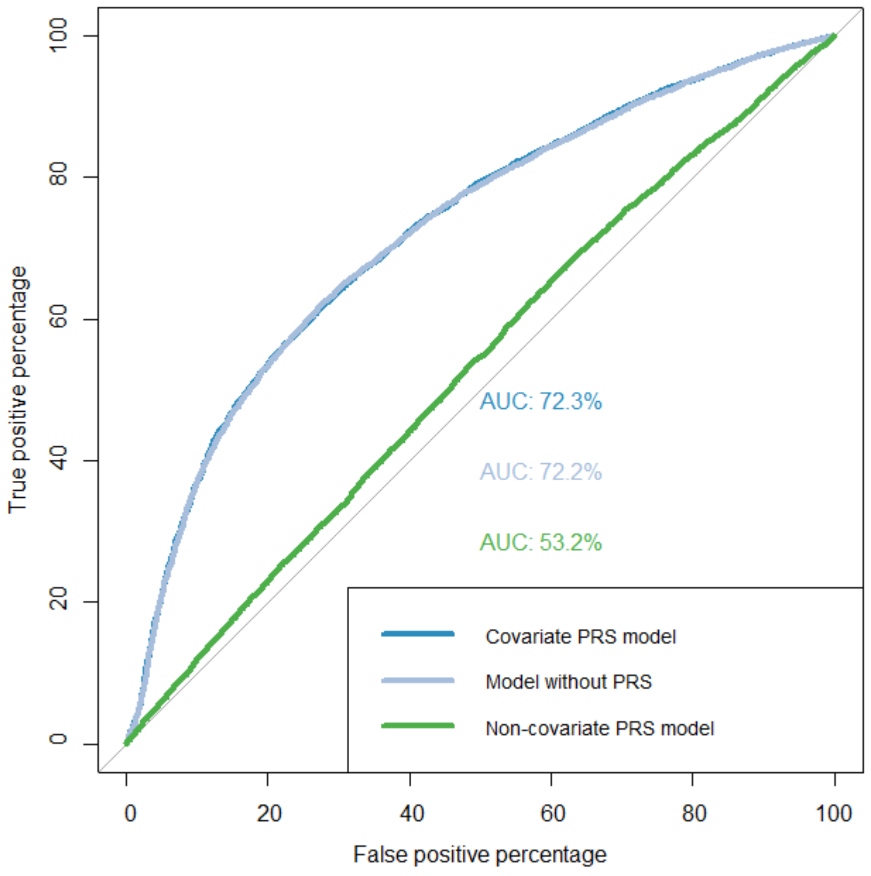

AUC: 72.3%
AUC: 72.2%
AUC: 53.2%
Covariate PRS model
Model without PRS
Non-covariate PRS model
True positive percentage
False positive percentage
0
20
40
60
80
100

C)

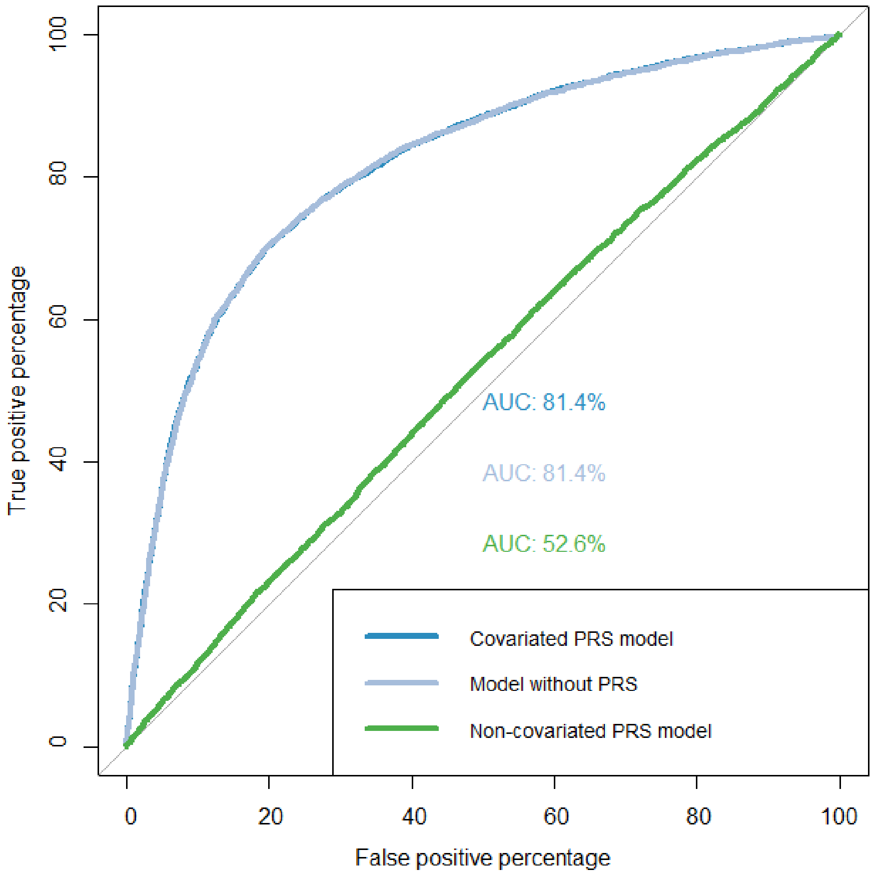

Figure S1. Areas under the curve (AUC) of the global SCZ PRS prediction for COVID-19 a) case/control, **b)** symptomatic/asymptomatic, and **c)** hospitalization/no hospitalization in the total sample.

Banner Scourge

Scourge Cohort Group

Javier Abellan[16,17]; René Acosta-Isaac[18]; Jose María Aguado[19,20,21,22]; Carlos Aguilar[23]; Sergio Aguilera-Albesa[24,25]; Abdolah Ahmadi Sabbagh[26]; Jorge Alba[27]; Sergiu Albu[28,29,30]; Karla A.M. Alcalá-Gallardo[31]; Julia Alcoba-Florez[32]; Sergio Alcolea Batres[33]; Holmes Rafael Algarin-Lara[34,35]; Virginia Almadana[36]; Julia Almeida[37,38,39,40]; Berta Almoguera[41,5]; María R. Alonso[42]; Nuria Alvarez[42]; Yady Álvarez-Benítez[34,35]; Felipe Álvarez-Navia[43,44]; Rodolfo Alvarez-Sala Walther[33]; Álvaro Andreu-Bernabeu[45,21]; Maria Rosa Antonijoan[46]; Eunate Arana-Arri[47,48]; Carlos Aranda[49,50]; Celso Arango[45,51,21]; Carolina Araque[52,53]; Nathalia K. Araujo[54]; Izabel M.T. Araujo[55]; Ana C. Arcanjo[56,57,58]; Ana Arnaiz[6,7,8]; Francisco Arnalich Fernández[59]; María J. Arranz[60]; José Ramon Arribas Lopez[59]; Maria-Jesus Artiga[61]; Yubelly Avello-Malaver[62]; Carmen Ayuso[41,5]; Ana María Baldion[62]; Belén Ballina Martín[26]; Raúl C. Baptista-Rosas[63,64,65]; Andrea Barranco-Díaz[35]; María Barreda- Sánchez[66,67] Viviana Barrera-Penagos[62]; Moncef Belhassen-Garcia[68,44]; Enrique Bernal[66]; David Bernal-Bello[69]; Joao F. Bezerra[70]; Marcos A.C. Bezerra[71]; Natalia Blanca-López[72]; Rafael Blancas[73]; Lucía Boix-Palop[74]; Alberto Borobia[75]; Elsa Bravo[76]; María Brion[77,78]; Óscar Brochado-Kith[79,22]; Ramón Brugada[80,81,78,82]; Matilde Bustos[83]; Alfonso Cabello[84]; Juan J. Caceres-Agra[85]; Esther Calbo[86]; Enrique J. Calderón[87,88,89]; Shirley Camacho[90]; Marcela C. Campos[56]; Yolanda Cañadas[50]; Cristina Carbonell[43,44]; Servando Cardona-Huerta[9]; Antonio Augusto F. Carioca[91]; Maria Sanchez Carpintero[49,50]; Carlos Carpio Segura[33]; Thássia M.T. Carratto[92]; José Antonio Carrillo-Avila[93]; Maria C.C. Carvalho[94]; Carlos Casasnovas[95,96,5]; Luis Castano[47,97,5,98,99]; Carlos F. Castaño[49,50]; Jose E. Castelao[100]; Aranzazu Castellano Candalija[101]; María A. Castillo[90]; Francisco C. Ceballos[79]; Jessica G. Chaux[53]; Walter G. Chaves- Santiago[102,53]; Sylena Chiquillo-Gómez[34,35]; Marco A. Cid-Lopez[31]; Oscar Cienfuegos-Jimenez[9]; Rosa Conde-Vicente[103]; M. Lourdes Cordero-Lorenzana[104]; Dolores Corella[105,106]; Almudena Corrales[12,13]; Jose L. Cortes-Sanchez[9,107]; Marta Corton[41,5]; Tatiana X. Costa[108]; Raquel Cruz[2,5]; Marina S. Cruz[54]; Luisa Cuesta[109]; Gabriela C.R. Cunha[110]; Gabriela V. da Silva[55]; David Dalmau[111,86]; Raquel C.S. Dantas-Komatsu[54]; M. Teresa Darnaude[112]; Raimundo de Andrés[113]; Jéssica N.G. de Araújo[114]; Carmen de Juan[115]; Juan De la Cruz Troca[116,117,88]; Carmen de la Horra[89]; Ana B. de la Hoz[47]; Alba De Martino-Rodríguez[118,119]; Julianna Lys de Sousa Alves Neri[120]; Victor del Campo-Pérez[121]; Juan Delgado-Cuesta[122]; Covadonga M. Diaz-Caneja[45,51,21]; Anderson Díaz-Pérez[35]; Aranzazu Diaz de Bustamante[112]; Beatriz Dietl[86]; Silvia Diz-de Almeida[2,5]; Manoella do Monte Alves[123,124]; Elena Domínguez-Garrido[125]; Katiusse A. dos Santos[94]; Alice M. Duarte[55]; Jose Echave-Sustaeta[126]; Rocío Eiros[127]; César O. Enciso-Olivera[52,53]; Gabriela Escudero[128]; Pedro Pablo España[129]; Gladys Mercedes Estigarribia Sanabria[130]; María Carmen Fariñas[6,7,8]; Marianne R. Fernandes[131,132]; Ramón Fernández[6,133]; Lidia Fernandez-Caballero[41,5]; Ana Fernández-Cruz[134]; María J. Fernandez-Nestosa[135]; Uxía Fernández-Robelo[136]; Amanda Fernández-Rodríguez[79,22]; Marta Fernández-Sampedro[6,7,8]; Ruth Fernández-Sánchez[41,5]; Tania Fernández-Villa[137]; Silvia Fernández Ferrero[26]; Yolanda Fernández Martínez[26]; Carmen Fernández Capitán[101]; Patricia Flores-Pérez[138]; Vicente Friaza[88,89]; Lácides Fuenmayor-Hernández[35]; Marta Fuertes Núñez[26]; Victoria Fumadó[139]; Ignacio Gadea[140]; Lidia Gagliardi[49,50]; Manuela Gago-Domínguez[3,4]; Natalia Gallego[10]; Cristina Galoppo[141]; Inés García[41,5]; Mercedes García[49,50]; Leticia García[49,50]; Carlos Garcia-Cerrada[16,17,5,142]; Aitor García-de-Vicuña[47,143]; Josefina Garcia-García[66]; Irene García-García[75]; Carmen García-Ibarbia[6,7,8]; Andrés C. García-Montero[144]; Ana García-Soidán[145]; Elisa García-Vázquez[66]; María Carmen García Torrejón[146,17]; Emiliano Garza-Frias[9]; Angela Gentile[141]; Belén Gil-

Fournier[147]; Javier Gómez-Arrue[118,119]; Mario Gómez-Duque[102,53]; Luis Gómez Carrera[33]; María Gómez García[3]; Ángela Gómez Sacristán[148]; Anna González-Neira[42]; Javier González-Peñas[45,21,51]; Manuel Gonzalez-Sagrado[103]; Beatriz González Álvarez[118,119]; Fernan Gonzalez Bernaldo de Quirós[149]; Hugo Gonzalo Benito[150]; Oscar Gorgojo-Galindo[151]; Miguel Górgolas[84]; Florencia Guaragna[141]; Genilson P. Guegel[152]; Beatriz Guillen-Guio[12]; Encarna Guillen-Navarro[66,153,154,155]; Pablo Guisado-Vasco[126]; Juan F. Gutiérrez-Bautista[156]; Luz D. Gutierrez-Castañeda[157,53]; Sarah Heili-Frades[158]; Estefania Hernandez[159]; Luis D. Hernandez-Ortega[160,161]; Guillermo Hernández-Pérez[43]; Rebeca Hernández-Vaquero[162]; Cristina Hernández Moro[26]; Belen Herraez[42]; M. Teresa Herranz[66]; María Herrera[49,50]; María José Herrero[163,164]; Antonio Herrero-Gonzalez[165]; Juan P. Horcajada[166,167,29,168,22]; Natale Imaz-Ayo[47]; Maider Intxausti-Urrutibeaskoa[169]; María Íñiguez[170]; Rafael H. Jacomo[171]; Rubén Jara[66]; Perez Maria Jazmin[141]; Ángel Jiménez[49,50]; Pilar Jiménez[156]; Ignacio Jiménez-Alfaro[172]; María A. Jimenez-Sousa[79,22]; Iolanda Jordan[173,174,88]; Rocío Laguna-Goya[175,176]; Daniel Laorden[33]; María Lasa-Lazaro[175,176]; María Claudia Lattig[90,177]; Ailen Lauriente[141]; Anabel Liger Borja[178]; Lucía Llanos[179]; Amparo López-Bernús[43,44]; Esther Lopez-Garcia[116,117,88,180]; Rosario Lopez-Rodriguez[41,5]; Miguel A. López-Ruz[181,182,183]; Eduardo López Granados[184,185,5]; Leonardo Lorente[186]; José E. Lozano[187]; María Lozano-Espinosa[178]; Andre D. Luchessi[188]; Ignacio Mahillo[189,190,13]; Esther Mancebo[175,176]; Carmen Mar[129]; Cristina Marcelo Calvo[101]; Miguel Marcos[43,44]; Alba Marcos-Delgado[191]; Alicia Marín Candon[75]; Pablo Mariscal Aguilar[33]; María M. Martín[192]; María Dolores Martín[193]; Vicente Martín[191,88]; Marta Martin-Fernandez[194]; Caridad Martín-López[178]; José-Ángel Martín-Oterino[43,44]; Laura Martin-Pedraza[72]; María Martín-Vicente[79]; Amalia Martinez[195]; Ricardo Martínez[159]; Juan José Martínez[96,5]; Silvia Martínez[6,8]; Eleno Martínez-Aquino[196]; Óscar Martínez-González[197]; Iciar Martinez-Lopez[198,199]; Oscar Martinez-Nieto[62,177]; Pedro Martinez-Paz[150]; Angel Martinez-Perez[200]; Andrea Martínez-Ramas[41,5]; Michel F. Martinez-Resendez[9]; Violeta Martínez Robles[26]; Laura Marzal[41,5]; Juliana F. Mazzeu[201,202,203]; Jeane F.P. Medeiros[54]; Kelliane A. Medeiros[204,205]; Francisco J. Medrano[87,88,89]; Xose M. Meijome[206,207]; Natalia Mejuto-Montero[208]; Ana Méndez-Echevarria[209]; Humberto Mendoza Charris[76,35]; Eleuterio Merayo Macías[210]; Fátima Mercadillo[211]; Arieh R. Mercado-Sesma[160,161]; Pablo Minguez[41,5]; Antonio J J. Molina[191,88]; Elena Molina-Roldán[212]; Juan José Montoya[159]; Vitor M.S. Moraes[92]; Patricia Moreira-Escriche[115]; Xenia Morelos-Arnedo[76,35]; Antonio Moreno-Docón[66]; Junior Moreno-Escalante[35]; Victor Moreno Cuerda[16,17]; Alberto Moreno Fernández[101]; Rubén Morilla[89,213]; Patricia Muñoz García[214,13,21]; Pablo Neira[141]; Julian Nevado[5,10,11]; Israel Nieto-Gañán[145]; Joana F.R. Nunes[56]; Rocio Nuñez- Torres[42]; Antònia Obrador-Hevia[215,216]; J. Gonzalo Ocejo-Vinyals[6,8]; Virginia Olivar[141]; Silviene F. Oliveira[201,217,218,219,220]; Lorena Ondo[41,5]; Alberto Orfao[37,38,39,40]; Luis Ortega[221]; Eva Ortega-Paino[61]; Fernando Ortiz-Flores[6,8]; Rocio Ortiz-Lopez[222,9]; José A. Oteo[27,170]; Harry Pachajoa[223,224]; Manuel Pacheco[159]; Fredy Javier Pacheco-Miranda[35]; Irene Padilla Conejo[26]; Sonia Panadero-Fajardo[93]; Mara Parellada[45,51,21]; Roberto Pariente-Rodríguez[145]; Estela Paz-Artal[175,176,225]; Germán Peces-Barba[226,13]; Miguel S. Pedromingo Kus[227]; Celia Perales[140]; Patricia Perez[228]; César Pérez[229]; Gustavo Perez-de-Nanclares[47,76]; Felipe Pérez-García[230,231]; Patricia Pérez-Matute[170]; Alexandra Pérez-Serra[80,78]; M. Elena Pérez-Tomás[66]; Teresa Perucho[232]; Lisbeth A. Pichardo[26]; Susana M.T. Pinho[204,233,234]; Mel·lina Pinsach-Abuin[80,78]; Luz Adriana Pinzón[102,53]; Guillermo Pita[42]; Francesc Pla-Junca[235,5]; Laura Planas-Serra[96,5]; Ericka N. Pompa-Mera[236]; Gloria L. Porras-Hurtado[159]; Aurora Pujol[96,5,237]; María Eugenia Quevedo Chávez[34,35]; Maria Angeles Quijada[46,238]; Inés Quintela[3]; Diana Ramirez-Montaño[239]; Soraya Ramiro León[147]; Pedro Rascado Sedes[240]; Delia Recalde[118,119]; Emma Recio-Fernández[170]; Salvador Resino[79,22]; Adriana P. Ribeiro[204,205,234]; Carlos S. Rivadeneira-Chamorro[53]; Diana Roa-Agudelo[62]; Montserrat Robelo Pardo[240]; Marilyn Johanna Rodriguez[53]; Fernando Rodriguez-Artalejo[116,117,88,180]; Marena Rodríguez-Ferrer[35]; Carlos Rodriguez-Gallego[241,15]; José A. Rodriguez-Garcia[26]; María A. Rodriguez-Hernandez[83]; Antonio Rodriguez-Nicolas[156]; Agustí Rodriguez-Palmero[242,96]; Emilio Rodríguez-Ruiz[240,4]; Paula A. Rodriguez-Urrego[62];

Belén Rodríguez Maya[16]; German Ezequiel Rodriguez Novoa[141]; Federico Rojo[243,40]; Andrea Romero-Coronado[35]; Filomeno Rondón García[26]; Lidia S. Rosa[244]; Antonio Rosales-Castillo[245]; Cladelis Rubio[246,247]; María Rubio Olivera[49,40]; Montserrat Ruiz[96,5]; Francisco Ruiz-Cabello[156,182,248]; Eva Ruiz-Casares[232]; Juan J. Ruiz-Cubillan[6,8]; Javier Ruiz-Hornillos[249,50,250]; Pablo Ryan[251,252,253]; Hector D. Salamanca[52,53]; Lorena Salazar-García[90]; Giorgina Gabriela Salgueiro Origlia[101]; Pedro-Luis Sánchez[127,44]; Clara Sánchez-Pablo[127]; Olga Sánchez-Pernaute[254]; Antonio J. Sánchez López[255]; María Concepción Sánchez Prados[33]; Javier Sánchez Real[26]; Jorge Sánchez Redondo[16,256]; Cristina Sancho-Sainz[169]; Anna Sangil[74]; Arnoldo Santos[229]; Ney P.C. Santos[131]; Agatha Schlüter[96,5]; Sonia Segovia[235,257,258]; Alex Serra-Llovich[259]; Fernando Sevil Puras[23]; Marta Sevilla Porras[5,10]; Miguel A. Sicolo[260,261]; Vivian N. Silbiger[188]; Nayara S. Silva[114]; Fabiola T.C. Silva[56]; Cristina Silván Fuentes[5]; Jordi Solé-Violán[262,13,263]; José Manuel Soria[200]; Jose V. Sorlí[105,106]; Renata R. Sousa[201]; Juan Carlos Souto[18]; Karla S.C. Souza[94]; Vanessa S. Souza[110]; John J. Sprockel[102,53]; José Javier Suárez-Rama[3]; David A. Suarez-Zamora[62]; Xiana Taboada-Fraga[208]; Eduardo Tamayo[264,151]; Alvaro Tamayo-Velasco[265]; Juan Carlos Taracido-Fernandez[165]; Nathali A.C. Tavares[266]; Carlos Tellería[118,119]; Jair Antonio Tenorio Castaño[5,10,11]; Alejandro Teper[141]; Juan Torres-Macho[267]; Lilian Torres-Tobar[53]; Ronald P. Torres Gutiérrez[227]; Jesús Troya[251]; Miguel Urioste[211]; Juan Valencia-Ramos[268]; Agustín Valido[36,269]; Juan Pablo Vargas Gallo[270,271]; Belén Varón[272]; Romero H.T. Vasconcelos[266]; Tomas Vega[273]; Santiago Velasco-Quirce[274]; Valentina Vélez-Santamaría[95,96]; Virginia Víctor[49,50]; Julia Vidán Estévez[26]; Miriam Vieitez-Santiago[6,8]; Carlos Vilches[275]; Lavinia Villalobos[26]; Felipe Villar[226]; Judit Villar-Garcia[276,277,278]; Cristina Villaverde[41,5]; Pablo Villoslada-Blanco[170]; Ana Virseda-Berdices[79]; Zuleima Yáñez[35]; Antonio Zapatero-Gaviria[279]; Ruth Zarate[280]; Sandra Zazo[243]; Miguel López de Heredia[5]; Ingrid Mendes[5]; Rocío Moreno[5]; Esther Sande[5,2]; Carlos Flores[12,13,14,15]; José A. Riancho[6,7,8]; Augusto Rojas-Martinez[9]; Pablo Lapunzina[5,10,11]; Angel Carracedo[2,3,4,5]

Scourge Cohort Group's filiations (436 members)

2. Centro Singular de Investigación en Medicina Molecular y Enfermedades Crónicas (CIMUS), Universidade de Santiago de Compostela, Santiago de Compostela, Spain
3. Fundación Pública Galega de Medicina Xenómica, Sistema Galego de Saúde (SERGAS) Santiago de Compostela, Spain
4. Grupo de Genética. Instituto de Investigación Sanitaria de Santiago (IDIS), Santiago de Compostela, Spain
5. Centro de Investigación Biomédica en Red de Enfermedades Raras (CIBERER-ISCIII), Instituto de Salud Carlos III, Madrid, Spain
6. IDIVAL, Santander, Spain
7. Universidad de Cantabria, Santander, Spain
8. Hospital U M Valdecilla, Santander, Spain
9. Tecnologico de Monterrey, Escuela de Medicina y Ciencias de la Salud and Hospital San Jose TecSalud, Monterrey, Mexico
10. Instituto de Genética Médica y Molecular (INGEMM), Hospital Universitario La Paz-IDIPAZ, Madrid, Spain
11. ERN-ITHACA-European Reference Network
12. Research Unit, Hospital Universitario N.S. de Candelaria, Santa Cruz de Tenerife, Spain
13. Centro de Investigación Biomédica en Red de Enfermedades Respiratorias (CIBERES), Instituto de Salud Carlos III, Madrid, Spain

14. Genomics Division, Instituto Tecnológico y de Energías Renovables, Santa Cruz de Tenerife, Spain
15. Department of Clinical Sciences, University Fernando Pessoa Canarias, Las Palmas de Gran Canaria, Spain
16. Hospital Universitario Mostoles, Medicina Interna, Madrid, Spain
17. Universidad Francisco de Vitoria, Madrid, Spain
18. Haemostasis and Thrombosis Unit, Hospital de la Santa Creu i Sant Pau, IIB Sant Pau, Barcelona, Spain
19. Unit of Infectious Diseases, Hospital Universitario 12 de Octubre, Instituto de Investigación Sanitaria Hospital 12 de Octubre (imas12), Madrid, Spain
20. Spanish Network for Research in Infectious Diseases (REIPI RD16/0016/0002), Instituto de Salud Carlos III, Madrid, Spain
21. School of Medicine, Universidad Complutense, Madrid, Spain
22. Centro de Investigación Biomédica en Red de Enfermedades Infecciosas (CIBERINFEC), Instituto de Salud Carlos III, Madrid, Spain
23. Hospital General Santa Bárbara de Soria, Soria, Spain
24. Pediatric Neurology Unit, Department of Pediatrics, Navarra Health Service Hospital, Pamplona, Spain
25. Navarra Health Service, NavarraBioMed Research Group, Pamplona, Spain
26. Complejo Asistencial Universitario de León, León, Spain
27. Hospital Universitario San Pedro, Infectious Diseases Department, Logroño, Spain
28. Fundación Institut Guttmann, Institut Universitari de Neurorehabilitació adscrit a la UAB, Hospital de Neurorehabilitació, Barcelona, Spain
29. Universitat Autònoma de Barcelona (UAB), Barcelona, Spain
30. Fundació Institut d'Investigació en Ciències de la Salut Germans Trias i Pujol, Barcelona, Spain
31. Hospital General de Occidente, Guadalajara, Mexico
32. Microbiology Unit, Hospital Universitario N. S. de Candelaria, Santa Cruz de Tenerife, Spain
33. Hospital Universitario La Paz-IDIPAZ, Servicio de Neumología, Madrid, Spain
34. Camino Universitario Adelita de Char, Mired IPS, Barranquilla, Colombia
35. Universidad Simón Bolívar, Facultad de Ciencias de la Salud, Barranquilla, Colombia
36. Hospital Universitario Virgen Macarena, Neumología, Seville, Spain
37. Departamento de Medicina, Universidad de Salamanca, Salamanca, Spain
38. Centro de Investigación del Cáncer (IBMCC) Universidad de Salamanca - CSIC, Salamanca, Spain
39. Biomedical Research Institute of Salamanca (IBSAL) Salamanca, Spain
40. Centre for Biomedical Network Research on Cancer (CIBERONC), Instituto de Salud Carlos III, Madrid, Spain
41. Department of Genetics & Genomics, Instituto de Investigación Sanitaria-Fundación Jiménez Díaz University Hospital - Universidad Autónoma de Madrid (IIS-FJD, UAM), Madrid, Spain
42. Spanish National Cancer Research Centre, Human Genotyping-CEGEN Unit, Madrid, Spain
43. Hospital Universitario de Salamanca-IBSAL, Servicio de Medicina Interna, Salamanca, Spain
44. Universidad de Salamanca, Salamanca, Spain
45. Department of Child and Adolescent Psychiatry, Institute of Psychiatry and Mental Health, Hospital General Universitario Gregorio Marañón (IiSGM), Madrid, Spain

46. Clinical Pharmacology Service, Hospital de la Santa Creu i Sant Pau, IIB Sant Pau, Barcelona, Spain

47. Biocruces Bizkai HRI, Barakaldo, Bizkaia, Spain

48. Cruces University Hospital, Osakidetza, Barakaldo, Bizkaia, Spain

49. Hospital Infanta Elena, Valdemoro, Madrid, Spain

50. Instituto de Investigación Sanitaria-Fundación Jiménez Díaz University Hospital - Universidad Autónoma de Madrid (IIS-FJD, UAM), Madrid, Spain

51. Centro de Investigación Biomédica en Red de Salud Mental (CIBERSAM), Instituto de Salud Carlos III, Madrid, Spain

52. Fundación Hospital Infantil Universitario de San José, Bogotá, Colombia

53. Fundación Universitaria de Ciencias de la Salud, Bogotá, Colombia

54. Universidade Federal do Rio Grande do Norte, Programa de Pós-graduação em Ciências da Saúde, Natal, Brazil

55. Universidade Federal do Rio Grande do Norte, Departamento de Medicina Clínica, Natal, Brazil

56. Departamento de Genética e Morfologia, Instituto de Ciências Biológicas, Universidade de Brasília, Brasilia, Brazil

57. Colégio Marista de Brasilia, Brazil

58. Associação Brasileira de Educação e Cultura, Brazil

59. Hospital Universitario La Paz-IDIPAZ, Servicio de Medicina Interna, Madrid, Spain

60. Fundació Docència I Recerca Mutua Terrassa, Barcelona, Spain

61. Spanish National Cancer Research Center, CNIO Biobank, Madrid, Spain

62. Fundación Santa Fe de Bogota, Departamento Patologia y Laboratorios, Bogotá, Colombia

63. Hospital General de Occidente, Zapopan, Jalisco, Mexico

64. Centro Universitario de Tonalá, Universidad de Guadalajara, Tonalá, Jalisco, Mexico

65. Centro de Investigación Multidisciplinario en Salud, Universidad de Guadalajara, Tonalá, Jalisco, Mexico

66. Instituto Murciano de Investigación Biosanitaria (IMIB-Arrixaca), Murcia, Spain

67. Universidad Católica San Antonio de Murcia (UCAM), Murcia, Spain

68. Hospital Universitario de Salamanca-IBSAL, Servicio de Medicina Interna-Unidad de Enfermedades Infecciosas, Salamanca, Spain

69. Hospital Universitario de Fuenlabrada, Department of Internal Medicine, Madrid, Spain

70. Escola Tecnica de Saúde, Laboratorio de Vigilancia Molecular Aplicada, Pará, Brazil

71. Federal University of Pernambuco, Genetics Postgraduate Program, Recife, PE, Brazil

72. Hospital Universitario Infanta Leonor, Servicio de Alergia, Madrid, Spain

73. Hospital Universitario del Tajo, Servicio de Medicina Intensiva, Aranjuez, Spain

74. Hospital Universitario Mutua Terrassa, Barcelona, Spain

75. Hospital Universitario La Paz-IDIPAZ, Servicio de Farmacología, Madrid, Spain

76. Alcaldía de Barranquilla, Secretaría de Salud, Barranquilla, Colombia

77. Instituto de Investigación Sanitaria de Santiago (IDIS), Xenética Cardiovascular, Santiago de Compostela, Spain

78. Centro de Investigación Biomédica en Red de Enfermedades Cardiovasculares (CIBERCV), Instituto de Salud Carlos III, Madrid, Spain

79. Unidad de Infección Viral e Inmunidad, Centro Nacional de Microbiología (CNM), Instituto de Salud Carlos III (ISCIII), Madrid, Spain
80. Cardiovascular Genetics Center, Institut d'Investigació Biomèdica Girona (IDIBGI), Girona, Spain
81. Medical Science Department, School of Medicine, University of Girona, Girona, Spain
82. Hospital Josep Trueta, Cardiology Service, Girona, Spain
83. Institute of Biomedicine of Seville (IBiS), Consejo Superior de Investigaciones Científicas (CSIC)- University of Seville- Virgen del Rocio University Hospital, Seville, Spain
84. Division of Infectious Diseases, Instituto de Investigación Sanitaria-Fundación Jiménez Díaz University Hospital - Universidad Autónoma de Madrid (IIS-FJD, UAM), Madrid, Spain
85. Intensive Care Unit, Hospital Universitario Insular de Gran Canaria, Las Palmas de Gran Canaria, Spain
86. Hospital Universitario Mutua Terrassa, Terrassa, Spain
87. Departemento de Medicina, Hospital Universitario Virgen del Rocío,Universidad de Sevilla, Seville, Spain
88. Centre for Biomedical Network Research on Epidemiology and Public Health (CIBERESP), Instituto de Salud Carlos III, Madrid, Spain
89. Instituto de Biomedicina de Sevilla, Seville, Spain
90. Universidad de los Andes, Facultad de Ciencias, Bogotá, Colombia
91. University of Fortaleza (UNIFOR), Department of Nutrition. Fortaleza, Brazil
92. Departamento de Química, Faculdade de Filosofia, Ciências e Letras de Ribeirão Preto, Universidade de São Paulo, Brazil
93. Andalusian Public Health System Biobank, Granada, Spain
94. Universidade Federal do Rio Grande do Norte, Programa de Pós-Graduação em Ciências Farmacêuticas, Natal, Brazil
95. Neuromuscular Unit, Neurology Department, Hospital Universitari de Bellvitge, L'Hospitalet de Llobregat (Barcelona), Spain
96. Bellvitge Biomedical Research Institute (IDIBELL), Neurometabolic Diseases Laboratory, L'Hospitalet de Llobregat, Spain
97. Osakidetza, Cruces University Hospital, Barakaldo, Bizkaia, Spain
98. Centre for Biomedical Network Research on Diabetes and Metabolic Associated Diseases (CIBERDEM), Instituto de Salud Carlos III, Madrid, Spain
99. University of Pais Vasco, UPV/EHU, Bizkaia, Spain
100. Oncology and Genetics Unit, Instituto de Investigacion Sanitaria Galicia Sur, Xerencia de Xestion Integrada de Vigo-Servizo Galego de Saúde, Vigo, Spain
101. Hospital Universitario La Paz, Hospital Carlos III, Madrid, Spain
102. Hospital de San José, Sociedad de Cirugía de Bogota, Bogotá, Colombia
103. Hospital Universitario Río Hortega, Valladolid, Spain
104. Servicio de Medicina intensiva, Complejo Hospitalario Universitario de A Coruña (CHUAC), Sistema Galego de Saúde (SERGAS), A Coruña, Spain
105. Valencia University, Preventive Medicine Department, Valencia, Spain
106. Centre for Biomedical Network Research on Physiopatology of Obesity and Nutrition (CIBEROBN), Instituto de Salud Carlos III, Madrid, Spain
107. Otto von Guericke University, Departament of Microgravity and Translational Regenerative Medicine, Magdeburg, Germany

108. Maternidade Escola Janário Cicco, Natal, Brazil
109. Institute of Psychiatry and Mental Health, Hospital General Universitario Gregorio Marañón (IiSGM), Madrid, Spain
110. Programa de Pós Graduação em Ciências da Saúde, Faculdade de Medicina, Universidade de Brasília, Brasilia, Brazil
111. Fundació Docència I Recerca Mutua Terrassa, Terrassa, Spain
112. Hospital Universitario Mostoles, Unidad de Genética, Madrid, Spain
113. Internal Medicine Department, Instituto de Investigación Sanitaria-Fundación Jiménez Díaz University Hospital - Universidad Autónoma de Madrid (IIS-FJD, UAM), Madrid, Spain
114. Universidade Federal do Rio Grande do Norte, Pós-graduação em Biotecnologia - Rede de Biotecnologia do Nordeste (Renorbio), Natal, Brazil
115. Hospital Universitario Severo Ochoa, Servicio de Medicina Interna, Madrid, Spain
116. Department of Preventive Medicine and Public Health, School of Medicine, Universidad Autónoma de Madrid, Madrid, Spain
117. IdiPaz (Instituto de Investigación Sanitaria Hospital Universitario La Paz), Madrid, Spain
118. Instituto Aragonés de Ciencias de la Salud (IACS), Zaragoza, Spain
119. Instituto Investigación Sanitaria Aragón (IIS-Aragon), Zaragoza, Spain
120. Universidade Federal do Rio Grande do Norte, Programa de Pós Graduação em Nutrição, Natal, Brazil
121. Preventive Medicine Department, Instituto de Investigacion Sanitaria Galicia Sur, Xerencia de Xestion Integrada de Vigo-Servizo Galego de Saúde, Vigo, Spain
122. Hospital Universitario Virgen del Rocío, Servicio de Medicina Interna, Seville, Spain
123. Universidade Federal do Rio Grande do Norte, Departamento de Infectologia, Natal, Brazil
124. Hospital de Doenças Infecciosas Giselda Trigueiro, Rio Grande do Norte, Natal, Brazil
125. Unidad Diagnóstico Molecular. Fundación Rioja Salud, La Rioja, Spain
126. Hospital Universitario Quironsalud Madrid, Madrid, Spain
127. Hospital Universitario de Salamanca-IBSAL, Servicio de Cardiología, Salamanca, Spain
128. Hospital Universitario Puerta de Hierro, Servicio de Medicina Interna, Majadahonda, Spain
129. Biocruces Bizkaia Health Research Institute, Galdakao University Hospital, Osakidetza, Bizkaia, Spain
130. Instituto Regional de Investigación en Salud-Universidad Nacional de Caaguazú, Caaguazú, Paraguay
131. Universidade Federal do Pará, Núcleo de Pesquisas em Oncologia, Belém, Pará, Brazil
132. Hospital Ophir Loyola, Departamento de Ensino e Pesquisa, Belém, Pará, Brazil
133. Fundación Asilo San Jose, Santander, Spain
134. Unidad de Enfermedades Infecciosas, Servicio de Medicina Interna, Hospital Universitario Puerta de Hierro, Instituto de Investigación Sanitaria Puerta de Hierro - Segovia de Arana, Madrid, Spain
135. Universidad Nacional de Asunción, Facultad de Politécnica, Paraguay
136. Urgencias Hospitalarias, Complejo Hospitalario Universitario de A Coruña (CHUAC), Sistema Galego de Saúde (SERGAS), A Coruña, Spain
137. Grupo de Investigación en Interacciones Gen-Ambiente y Salud (GIIGAS) - Instituto de Biomedicina (IBIOMED), Universidad de León, León, Spain
138. Hospital Universitario Niño Jesús, Pediatrics Department, Madrid, Spain

139. Unitat de Malalties Infeccioses i Importades, Servei de Pediatría, Infectious and Imported Diseases, Pediatric Unit, Hospital Universitari Sant Joan de Deú, Barcelona, Spain
140. Microbiology Department, Instituto de Investigación Sanitaria-Fundación Jiménez Díaz University Hospital - Universidad Autónoma de Madrid (IIS-FJD, UAM), Madrid, Spain
141. Hospital de Niños Ricardo Gutierrez, Buenos Aires, Argentina
142. Universidad Francisco de Vitoria, Madrid,Spain
143. Osakidetza, Cruces University Hospital, Bizkaia, Spain
144. University of Salamanca, Biomedical Research Institute of Salamanca (IBSAL), Salamanca, Spain
145. Department of Immunology, IRYCIS, Hospital Universitario Ramón y Cajal, Madrid, Spain
146. Hospital Infanta Elena, Servicio de Medicina Intensiva, Valdemoro, Madrid, Spain
147. Hospital Universitario de Getafe, Servicio de Genética, Madrid, Spain
148. Pneumology Department, Hospital General Universitario Gregorio Marañón (iiSGM), Madrid, Spain
149. Ministerio de Salud Ciudad de Buenos Aires, Buenos Aires, Argentina
150. Hospital Clinico Universitario de Valladolid, Unidad de Apoyo a la Investigación, Valladolid, Spain
151. Universidad de Valladolid, Departamento de Cirugía, Valladolid, Spain
152. Secretaria Municipal de Saude de Apodi, Natal, Brazil
153. Sección Genética Médica - Servicio de Pediatría, Hospital Clínico Universitario Virgen de la Arrixaca, Servicio Murciano de Salud, Murcia, Spain
154. Departamento Cirugía, Pediatría, Obstetricia y Ginecología, Facultad de Medicina, Universidad de Murcia (UMU), Murcia, Spain
155. Grupo Clínico Vinculado, Centre for Biomedical Network Research on Rare Diseases (CIBERER), Instituto de Salud Carlos III, Madrid, Spain
156. Hospital Universitario Virgen de las Nieves, Servicio de Análisis Clínicos e Inmunología, Granada, Spain
157. Hospital Universitario Centro Dermatológico Federico Lleras Acosta, Bogotá, Colombia
158. Intermediate Respiratory Care Unit, Department of Pneumology, Instituto de Investigación Sanitaria-Fundación Jiménez Díaz University Hospital - Universidad Autónoma de Madrid (IIS-FJD, UAM), Madrid, Spain
159. Clinica Comfamiliar Risaralda, Pereira, Colombia
160. Centro Universitario de Tonalá, Universidad de Guadalajara, Guadalajara, Mexico
161. Centro de Investigación Multidisciplinario en Salud, Universidad de Guadalajara, Guadalajara, Mexico
162. Unidad de Cuidados, Intensivos Hospital Clínico Universitario de Santiago (CHUS), Sistema Galego de Saúde (SERGAS), Santiago de Compostela, Spain
163. IIS La Fe, Plataforma de Farmacogenética, Valencia, Spain
164. Universidad de Valencia, Departamento de Farmacología, Valencia, Spain
165. Data Analysis Department, Instituto de Investigación Sanitaria-Fundación Jiménez Díaz University Hospital - Universidad Autónoma de Madrid (IIS-FJD, UAM), Madrid, Spain
166. Hospital del Mar, Infectious Diseases Service, Barcelona, Spain
167. Institut Hospital del Mar d’Investigacions Mèdiques (IMIM), Barcelona, Spain
168. CEXS-Universitat Pompeu Fabra, Spanish Network for Research in Infectious Diseases (REIPI), Barcelona, Spain

169. Biocruces Bizkaia Health Research Institute, Basurto University Hospital, Osakidetza, Bizkaia, Spain
170. Infectious Diseases, Microbiota and Metabolism Unit, Center for Biomedical Research of La Rioja (CIBIR), Logroño, Spain
171. Sabin Medicina Diagnóstica, Brazil
172. Opthalmology Department, Instituto de Investigación Sanitaria-Fundación Jiménez Díaz University Hospital - Universidad Autónoma de Madrid (IIS-FJD, UAM), Madrid, Spain
173. Hospital Sant Joan de Deu,Pediatric Critical Care Unit, Barcelona, Spain
174. Paediatric Intensive Care Unit, Agrupación Hospitalaria Clínic-Sant Joan de Déu, Esplugues de Llobregat, Barcelona, Spain
175. Hospital Universitario 12 de Octubre, Department of Immunology, Madrid, Spain
176. Instituto de Investigación Sanitaria Hospital 12 de Octubre (imas12), Transplant Immunology and Immunodeficiencies Group, Madrid, Spain
177. SIGEN Alianza Universidad de los Andes - Fundación Santa Fe de Bogotá, Bogotá, Colombia
178. Hospital General de Segovia, Medicina Intensiva, Segovia, Spain
179. Clinical Trials Unit, Instituto de Investigación Sanitaria-Fundación Jiménez Díaz University Hospital - Universidad Autónoma de Madrid (IIS-FJD, UAM), Madrid, Spain
180. IMA-Food Institute, CEI UAM+CSIC, Madrid, Spain
181. Hospital Universitario Virgen de las Nieves, Servicio de Enfermedades Infecciosas, Granada, Spain
182. Instituto de Investigación Biosanitaria de Granada (ibs GRANADA), Granada, Spain
183. Universidad de Granada, Departamento de Medicina, Granada, Spain
184. Hospital Universitario La Paz-IDIPAZ, Servicio de Inmunología, Madrid, Spain
185. La Paz Institute for Health Research (IdiPAZ), Lymphocyte Pathophysiology in Immunodeficiencies Group, Madrid, Spain
186. Intensive Care Unit, Hospital Universitario de Canarias, La Laguna, Spain
187. Dirección General de Salud Pública, Consejería de Sanidad, Junta de Castilla y León, Valladolid, Spain
188. Universidade Federal do Rio Grande do Norte, Departamento de Analises Clinicas e Toxicologicas, Natal, Brazil
189. Fundación Jiménez Díaz, Epidemiology, Madrid, Spain
190. Universidad Autónoma de Madrid, Department of Medicine, Madrid, Spain
191. Instituto de Biomedicina (IBIOMED), Universidad de León, León, Spain
192. Intensive Care Unit, Hospital Universitario N. S. de Candelaria, Santa Cruz de Tenerife, Spain
193. Preventive Medicine Department, Instituto de Investigación Sanitaria-Fundación Jiménez Díaz University Hospital - Universidad Autónoma de Madrid (IIS-FJD, UAM), Madrid, Spain
194. Universidad de Valladolid, Departamento de Medicina, Valladolid, Spain
195. Hospital Universitario Infanta Leonor, Servicio de Medicina Intensiva, Madrid, Spain
196. Servicio de Medicina Interna, Sanatorio Franchin, Buenos Aires, Argentina
197. Hospital Universitario del Tajo, Servicio de Medicina Intensiva, Toledo, Spain
198. Unidad de Genética y Genómica Islas Baleares, Islas Baleares, Spain
199. Hospital Universitario Son Espases, Unidad de Diagnóstico Molecular y Genética Clínica, Islas Baleares, Spain

200. Genomics of Complex Diseases Unit, Research Institute of Hospital de la Santa Creu i Sant Pau, IIB Sant Pau, Barcelona, Spain

201. Faculdade de Medicina, Universidade de Brasília, Brasilia, Brazil

202. Programa de Pós-Graduação em Ciências Médicas, Universidade de Brasília, Brasilia, Brazil

203. Programa de Pós-Graduação em Ciências da Saúde, Universidade de Brasília, Brasilia, Brazil

204. Hospital das Forças Armadas, Brazil

205. Exército Brasileiro, Brazil

206. Hospital El Bierzo, Gerencia de Asistencia Sanitaria del Bierzo (GASBI), Gerencia Regional de Salud (SACYL), Ponferrada, Spain

207. Grupo INVESTEN, Instituto de Salud Carlos III, Madrid, Spain

208. Unidad de Cuidados Intensivos, Complejo Universitario de A Coruña (CHUAC), Sistema Galego de Saúde (SERGAS), A Coruña, Spain

209. Hospital Universitario La Paz-IDIPAZ, Servicio de Pediatría, Madrid, Spain

210. Hospital El Bierzo, Unidad Cuidados Intensivos, León, Spain

211. Spanish National Cancer Research Centre, Familial Cancer Clinical Unit, Madrid, Spain

212. Instituto de Investigación Sanitaria San Carlos (IdISSC), Hospital Clínico San Carlos (HCSC), Madrid, Spain

213. Universidad de Sevilla, Departamento de Enfermería, Seville, Spain

214. Hospital General Universitario Gregorio Marañón (IiSGM), Madrid, Spain

215. Unidad de Genética y Genómica Islas Baleares, Unidad de Diagnóstico Molecular y Genética Clínica, Hospital Universitario Son Espases, Islas Baleares, Spain

216. Instituto de Investigación Sanitaria Islas Baleares (IdISBa), Islas Baleares, Spain

217. Programa de Pós-Graduação em Biologia Animal, Universidade de Brasília, Brasília, Brazil

218. Programa de Pós-Graduação em Ciências da Saúde, Universidade de Brasília, Brasília, Brazil

219. Programa de Pós-Graduação Profissional em Ensino de Biologia, Universidade de Brasília, Brasília, Brazil

220. Programa de Pós-Graduação em Ciências Médicas, Universidade de Brasília, Brasília, Brazil

221. Anatomía Patológica, Instituto de Investigación Sanitaria San Carlos (IdISSC), Hospital Clínico San Carlos (HCSC), Madrid, Spain

222. Tecnológico de Monterrey, Monterrey, Mexico

223. Centro de Investigación en Anomalías Congénitas y Enfermedades Raras (CIACER), Universidad Icesi

224. Departamento de Genetica, Fundación Valle del Lili

225. Universidad Complutense de Madrid, Department of Immunology, Ophthalmology and ENT, Madrid, Spain

226. Department of Neumology, Instituto de Investigación Sanitaria-Fundación Jiménez Díaz University Hospital - Universidad Autónoma de Madrid (IIS-FJD, UAM), Madrid, Spain

227. Hospital Nuestra Señora de Sonsoles, Ávila, Spain

228. Inditex, A Coruña, Spain

229. Intensive Care Department, Instituto de Investigación Sanitaria-Fundación Jiménez Díaz University Hospital - Universidad Autónoma de Madrid (IIS-FJD, UAM), Madrid, Spain

230. Hospital Universitario Príncipe de Asturias, Servicio de Microbiología Clínica, Madrid, Spain

231. Universidad de Alcalá de Henares, Departamento de Biomedicina y Biotecnología, Facultad de Medicina y Ciencias de la Salud, Madrid, Spain

232. GENYCA, Madrid, Spain
233. Marinha do Brasil, Brazil
234. Universidade de Brasília, Brasilia, Brazil
235. Neuromuscular Diseases Unit, Department of Neurology, Hospital de la Santa Creu i Sant Pau, Universitat Autònoma de Barcelona, Barcelona, Spain
236. Instituto Mexicano del Seguro Social (IMSS), Centro Médico Nacional Siglo XXI, Unidad de Investigación Médica en Enfermedades Infecciosas y Parasitarias, Mexico City, Mexico
237. Catalan Institution of Research and Advanced Studies (ICREA), Barcelona, Spain
238. Drug Research Centre, Institut d'Investigació Biomèdica Sant Pau, IIB-Sant Pau, Barcelona, Spain
239. Departamento de Genetica, Clinica imbanaco
240. Unidad de Cuidados Intensivos, Hospital Clínico Universitario de Santiago (CHUS), Sistema Galego de Saúde (SERGAS), Santiago de Compostela, Spain
241. Department of Immunology, Hospital Universitario de Gran Canaria Dr. Negrín, Las Palmas de Gran Canaria, Spain
242. University Hospital Germans Trias i Pujol, Pediatrics Department, Badalona, Spain
243. Department of Pathology, Biobank, Instituto de Investigación Sanitaria-Fundación Jiménez Díaz University Hospital - Universidad Autónoma de Madrid (IIS-FJD, UAM), Madrid, Spain
244. Faculdade de Ciências da Saúde, Universidade de Brasília, Brasilia, Brazil
245. Hospital Universitario Virgen de las Nieves, Servicio de Medicina Interna, Granada, Spain
246. Fundación Universitaria de Ciencias de la Salud, Grupo de Ciencias Básicas en Salud (CBS), Bogotá, Colombia
247. Sociedad de Cirugía de Bogotá, Hospital de San José, Bogotá, Colombia
248. Universidad de Granada, Departamento Bioquímica, Biología Molecular e Inmunología III, Granada, Spain
249. Hospital Infanta Elena, Allergy Unit, Valdemoro, Madrid, Spain
250. Faculty of Medicine, Universidad Francisco de Vitoria, Madrid, Spain
251. Hospital Universitario Infanta Leonor, Madrid, Spain
252. Complutense University of Madrid, Madrid, Spain
253. Gregorio Marañón Health Research Institute (IiSGM), Madrid, Spain
254. Reumathology Service, Instituto de Investigación Sanitaria-Fundación Jiménez Díaz University Hospital - Universidad Autónoma de Madrid (IIS-FJD, UAM), Madrid, Spain
255. Biobank, Puerta de Hierro-Segovia de Arana Health Research Institute, Madrid, Spain
256. Universidad Rey Juan Carlos, Madrid, Spain
257. The John Walton Muscular Dystrophy Research Centre, Newcastle University and Newcastle Hospitals NHS Foundation Trust, Newcastle upon Tyne, UK.
258. Neuromuscular Unit, Neuropediatrics Department, Institut de Recerca Sant Joan de Déu, Hospital Sant Joan de Déu, Spain
259. Fundació Docència i Recerca Mutua Terrassa, Terrassa, Spain
260. Casa de Saúde São Lucas, Natal, Brazil
261. Hospital Rio Grande, Rio Grande do Norte, Natal, Brazil
262. Intensive Care Unit, Hospital Universitario de Gran Canaria Dr. Negrín, Las Palmas de Gran Canaria, Spain
263. Universidad Fernando Pessoa Canarias, Las Palmas de Gran Canaria, Spain

264. Hospital Clinico Universitario de Valladolid, Servicio de Anestesiologia y Reanimación, Valladolid, Spain
265. Hospital Clinico Universitario de Valladolid, Servicio de Hematologia y Hemoterapia, Valladolid, Spain
266. Hospital Universitario Lauro Wanderley, Brazil
267. Hospital Universitario Infanta Leonor, Servicio de Medicina Interna, Madrid, Spain
268. University Hospital of Burgos, Burgos, Spain
269. Universidad de Sevilla, Seville, Spain
270. Fundación Santa Fe de Bogota, Instituto de servicios medicos de Emergencia y trauma, Bogotá, Colombia
271. Universidad de los Andes, Bogotá, Colombia
272. Quironprevención, A Coruña, Spain
273. Junta de Castilla y León, Consejería de Sanidad, Valladolid, Spain
274. Gerencia Atención Primaria de Burgos, Burgos, Spain
275. Immunogenetics-Histocompatibility group, Servicio de Inmunología, Instituto de Investigación Sanitaria Puerta de Hierro - Segovia de Arana, Madrid, Spain
276. Hospital del Mar, Department of Infectious Diseases, Barcelona, Spain
277. IMIM (Hospital del Mar Medical Research Institute, Institut Hospital del Mar d'Investigacions Mediques), Barcelona, Spain
278. Universitat Autònoma de Barcelona, Department of Medicine, Spain
279. Consejería de Sanidad, Comunidad de Madrid, Madrid, Spain
280. Centro para el Desarrollo de la Investigación Científica, Asunción, Paraguay
